# The respiratory microbiota during health and disease: a paediatric perspective

**DOI:** 10.15172/pneu.2015.6/656

**Published:** 2015-12-01

**Authors:** Ilan J. N. Koppen, Astrid A. T. M. Bosch, Elisabeth A. M. Sanders, Marlies A. van Houten, Debby Bogaert

**Affiliations:** 112 Department of Paediatric Immunology and Infectious Diseases Wilhelmina Children Hospital, University Medical Centre Utrecht, Lundlaan 6, 3584 EA Utrecht, the Netherlands; 212 Spaarne Gasthuis Academy, Hoofddorp and Haarlem, the Netherlands

**Keywords:** microbiome, microbiota, respiratory infections, children, paediatric, respiratory tract

## Abstract

Recent studies investigating the relationship between the microbiota and disease are demonstrating novel concepts that could significantly alter the way we treat disease and promote health in the future. It is suggested that the microbiota acquired during childhood may shape the microbial community and affect immunological responses for later life, and could therefore be important in the susceptibility towards disease. Several diseases, including asthma, pneumonia, and otitis media, are associated with changes in composition and diversity of the respiratory microbiota. This review summarises current literature, focusing on the composition and development of the respiratory microbiota in children and its relationship with respiratory diseases.

## 1. Introduction

Lower respiratory tract infections remain one of the leading causes of mortality in young children worldwide, accounting for 1.4 to 1.8 million deaths annually [1]. Upper respiratory tract infections contribute considerably to healthcare utilisation in the paediatric population. Otitis media (OM), in particular, is a major cause for healthcare visits and antibiotic prescriptions among children [2]. Respiratory tract infections are frequently caused by respiratory viruses and potentially pathogenic bacteria (including *Streptococcus pneumoniae, Haemophilus influenzae, Moraxella catarrhalis*, and *Staphylococcus aureus*), or a combination of both viruses and bacteria; however, asymptomatic carriage with these potential pathogens also commonly occurs [3,4]. This contradictory finding suggests that the pathophysiology of respiratory infections is more complex than previously assumed. Moreover, a growing body of evidence suggests that susceptibility to respiratory tract infections is influenced by a number of factors related to the microbial communities that reside in (and on) the human body and involves more than mere colonisation with potential pathogens.

Over the last decade it has become clear that humans live in symbiosis with their abundant resident microbes, with bacterial cells outnumbering human cells by an estimated factor of 10 [5–7]. Advances in culture-independent techniques have enabled us to identify and accurately detect species that could not be detected by culture-based methods in the past [8]. These advances in the field of molecular techniques, in particular metagenomics, have led to the definition of the human microbiota, a term that refers to the complex microbial ecosystem in and on our bodies. The human microbiota is assumed to play an important role in human health by protecting the host against invading pathogens, contributing to the development of both innate and adaptive immunity, and aiding host nutrition and metabolite digestion [5,9,10]. Strong associations between microbiota characteristics and disease susceptibility have been described for a wide spectrum of diseases (e.g. inflammatory bowel disease, asthma, psoriasis, and colorectal cancer) [5,9,11].

Investigating the role of the microbiota in health and disease is a relatively new, rapidly developing field of research. Since the human microbiota has been shown to be associated with susceptibility to a wide variety of diseases, it can be hypothesised that the microbiota of the respiratory tract is involved in containment of potential pathogens as well; therefore, changes in microbial composition of the respiratory tract may increase susceptibility to respiratory diseases. A better understanding of respiratory infection pathogenesis is needed to identify novel therapeutic possibilities. This review summarises current knowledge on the composition and diversity of the microbiota of the respiratory tract and its possible relationship with the development of respiratory diseases.

## 2. The microbiota of the respiratory tract from a health perspective

The mucosal surface of the respiratory tract is in contact with the outside world with every breath we take. The microbial communities that reside in the different niches of the respiratory tract differ significantly, which is most probably driven by the interplay between environmental influences (e.g. temperature, humidity, and oxygen saturation), anatomical factors, and epithelial characteristics and functions. Despite the significant differences between different niches along the respiratory tract, there is an overlap in microbial communities found in neighbouring niches, presumably as a consequence of anatomical adjacency and similar environmental conditions [12]. An overview of microbiota composition differences between these niches in the respiratory tract is discusssed.

The frontal part of the nose, the nares (nostrils), is anatomically located in a way that renders it highly susceptible to influences from the outside world. The microbiota of the nares harbours a very diverse bacterial community, including commensals and potential pathogens. In children, Gram-positive aerobes such as *Staphylococcus, Streptococcus* (mainly *Streptococcus salivarius* and *Streptococcus mitis*), *Dolosigranulum, Corynebacterium, Gemella, Granulicatella* spp., and (to a lesser extent) Gram-negative aerobes commonly found in adults such as *Moraxella, Haemophilus*, and *Neisseria* spp. have been described [13]. The nasopharynx, which connects the nose and oropharynx, is a well-known reservoir for potential pathogens that are able to cause respiratory tract infections: its microbiota composition resembles that of the nares, although Gram-negative and anaerobic bacteria are more predominant presumably due to the ecological circumstances [4]. The prevalence of the *Staphylococcaceae* family, including *S. aureus*, seems to be inversely correlated with prevalence of commensals of the *Corynebacteriaceae* and *Propionibacteriaceae* families [14]. This inverse relationship between specific commensals and potential pathogens might indicate that bacterial interactions play an important role in the composition of the microbiota in this niche.

Due to its anatomy and function, the oral cavity is strongly influenced by feeding. It harbours a highly diverse microbiota, characterised by *Streptococcus, Actinomyces, Gemella*, and *Veillonella* spp. There are many similarities and overlap between the oral microbiota composition and the microbiota of the nasopharynx and lungs [5,15].

Studying the lung microbiota is a relatively new field and sampling of healthy subjects for research purposes is difficult due to its invasive character. Although the lungs were classically believed to be sterile, recent culture-independent studies have shown otherwise [11,16]. The members of the lung microbiota resemble those of the upper respiratory tract microbiota, although the bacterial density is much lower [12]. It remains unclear, however, whether the microbiota as observed in the lower respiratory tract of healthy individuals resembles a true ecosystem, or if it is the result of contamination during sampling, or continuous microaspiration from the upper respiratory tract [17].

### 2.1 Development of the respiratory tract microbiota with age

**Figure 1:**
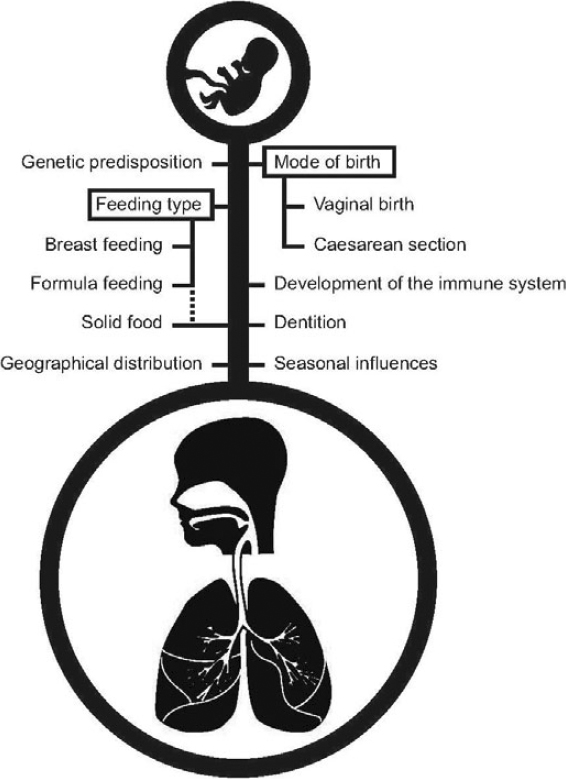
Schematic representation of internal and external drivers of development of the respiratory microbiota in the first years of life

Until recently, it was believed that the intrauterine environment was sterile. However, culture-independent techniques have challenged this dogma [18]. Birth is considered by many to be the main starting point of the establishment of the human microbiota [5,19,20]. During the first weeks of newborn life, the microbiota of different body sites, including the respiratory tract, will start to develop. This process is most likely dependent on internal factors such as genetic predisposition, and external factors—most notably, the maternal microbiota and mode of delivery [9,11] (Figure 1).

Mode of delivery is presumed to be of key importance in the initial development of the microbiota [5]. In vaginally delivered children, the first bacterial communities in the respiratory tract resemble the vaginal microflora from the mother (*Lactobacillus, Prevotella*, or *Sneathia* spp.) whereas children born by Caesarean section are predominantly colonised by skin-type bacteria such as *Staphylococcus, Corynebacterium*, and *Propionibacterium* spp. [5,20]. A comparable pattern of colonisation succession seems to take place in the microbiota of the skin, gut, and airways [20]. Mode of delivery, therefore, seems to determine the initial colonisation of all body sites of the newborn and is a potential driver for the niche-specific differentiation that occurs afterwards.

One of the first major encounters in early life is food; breastfeeding and formula-feeding impose selective effects on the composition of the intestinal microbiota of infants regarding the abundance of certain genera such as *Bifidobacterium* and *Lactobacillus* [5,7,19,21]. Part of this effect is ascribed to human milk oligosaccharides that stimulate the growth of *Bifidobacterium* spp. [5]. It is likely that feeding also shapes the microbiota of other niches, such as the respiratory niches. This is supported by a recent study by Biesbroek et al. [22], which showed that the nasopharyngeal microbiota composition differs significantly between breastfed and formula-fed young infants. In this study, breastfed children had significantly higher abundances of the Gram-positive commensals *Dolosigranulum* and *Corynebacterium* and decreased abundance of *Staphylococcus* spp. Even more interesting is that this difference in microbiota composition appeared to correlate with respiratory health: infants with *Dolosigranulum*- and *Corynebacterium*-dominated nasopharyngeal microbiota experienced decreased numbers of episodes of respiratory infections with wheezing or symptoms of respiratory infections in the consecutive months of life.

In general, the composition of the early microbiota seems to be determinative in shaping the microbiota later in life. In a recent study by our research group, we showed that respiratory profiles at 6 weeks of age were associated with stability and patterns of change over the first 24 months of life [23]. Moreover, stability of microbiota was inversely associated with symptoms of respiratory infections in the consecutive periods. Therefore, the composition of the early microbiota may play an important role in the stability and composition of the microbiota profile later on during childhood. Furthermore, maturation of the immune system will have an effect on, but inversely will also be influenced by, the microbiota composition overtime [4,24–29].

### 2.2 Dysbiosis of the microbiota

The discovery of penicillin by Alexander Fleming in 1928 has been crucial for current medical practice. Nowadays, children in developed countries generally receive between 10 and 20 courses of antibiotics before reaching adulthood [30]. Extensive antibiotic use has, however, led to the development of antibiotic resistance, which is a major reason for increased efforts on antibiotic stewardship worldwide. Additionally, antibiotic treatment is assumed to elicit major perturbations of the human microbiota [31–33]. In the gut, antibiotics cause a disruption of microbiota composition as well as a decrease in biodiversity [34]. The same profound effect of antibiotics has been described for the respiratory microbiota [35–40]. The consequences of a lower biodiversity are not yet fully understood although recent data show a strong association between decreased taxonomic richness and evenness of microbial communities and diseases such as necrotising enterocolitis and inflammatory bowel diseases [5]. Antibiotic use during infancy has also been associated with antibiotic consumption and risk of developing asthma and obesity over time [9,41,42]. More research is needed to elucidate the effects of antibiotic treatment regimens on the microbiota in both the short and long term.

Vaccines targeting respiratory pathogens are also considered to have an important impact on the respiratory microbiota composition. For example, the introduction of the pneumococcal conjugate vaccines has been shown to elicit a major effect on the composition of the respiratory microbiota. Although a significant decrease in colonisation with vaccine serotypes pneumococci was observed, the vacant niche was rapidly filled by non-vaccine serotypes [43,44]. In addition, pneumococcal conjugate vaccines have provoked an effect on the microbiota of the respiratory tract that transcends the elimination of *S. pneumoniae*. Culture-based studies showed that *S. aureus* and *H. influenzae* were persistently present in the nasopharynx of young children and their parents after the introduction of the vaccine, compared to pre-vaccination [43,45]. Through 16S-based sequencing, a higher inter-individual variability in microbiota composition and increased diversity of bacterial species were observed in immunised children at 12 months of age when compared to non-immunised children 1 month following a booster vaccination [46]. As expected, the composition of the nasopharyngeal microbiota of these vaccinated children was characterised by a decrease in pneumococcal presence; however, non-pneumococcal streptococcal, and anaerobic bacterial presence increased and an apparent higher abundance of *Haemophilus* and *Staphylococcus* was observed. Although these differences might display a temporary effect, the long-term health effects of these changes are not fully known and need to be further investigated.

### 2.3 Interspecies interaction

In addition to the presence or absence of certain bacteria, it is believed that bacterial interactions play an important role in pathogenesis of respiratory infections. For example, children with high levels of *Rothia, Gemella, Actinomyces, Veillonella*, and *Granulicatella* are less likely to be colonised with *S. pneumoniae* in the upper respiratory tract [35]. In contrast, pneumococcal carriage is negatively associated with the presence of bacteria including *S. salivarius, S. mitis*, and *S. aureus*, suggesting negative interactions between these species [37,47,48]. Moreover, carriage of species including *S. pneumoniae, H. influenzae*, or *M. catarrhalis* is associated with lower microbial diversity in upper respiratory tract flora [35,49]. It remains unclear whether microbial diversity is independently associated with respiratory health characteristics or merely relies on microbial community composition.

Besides bacterial interactions, viruses appear to play an important role in respiratory health in children. Viral infections often precede a bacterial superinfection [50]. Similar to asymptomatic carriage of potential pathogenic bacteria, respiratory viruses seem to inhabit the respiratory tract of healthy children frequently, forming the so-called virome [3,50,51]. Therefore interactions between viruses and bacteria are potentially of great significance in the phases preceding respiratory tract infections as well.

## 3. The microbiota during respiratory tract diseases

It has been hypothesised that the composition of the microbiota in the respiratory tract might be altered during respiratory diseases. We describe the literature regarding associations between microbial composition and upper and lower respiratory tract infections, cystic fibrosis (CF), bronchopulmonary dysplasia (BPD), and asthma.

### 3.1 Upper respiratory tract infectons

OM is one of the most common paediatric infectious diseases, affecting the majority of children in developed and developing countries [2,52]. It is a major reason for healthcare visits and antibiotic prescription in children [2,49,53].

OM is frequently associated with viral respiratory infections; however, it can also be caused by bacterial pathogens. The most commonly observed bacterial species in OM are *S. pneumoniae, H. influenzae*, and *M. catarrhalis* [39,54]. Mixed cultures with other species are also found, making it difficult to differentiate between causal and bystander effects.

Microbiota studies of the upper respiratory tract in relation to respiratory infections are scarce. However, Pettigrew and colleagues [35] observed that an increased presence of *Streptococcus* and *Haemophilus* was associated with a decreased presence of *Corynebacterium* and *Dolosigranulum* in the upper respiratory tract of children with a respiratory infection, suggesting a possible protective effect of the latter species in the development of upper respiratory tract infections. In another study, Hilty et al. [39] compared nasopharyngeal swabs of controls and infants with acute OM, and reported a significantly higher abundance of *Staphylococcaceae* in controls and a non-significant trend towards a higher abundance of *Moraxellaceae, Streptococcaceae*, and *Pasteurellaceae* in children with acute OM.

Nasal colonisation with potential otopathogens such *S. pneumoniae, H. influenzae*, and *M. catarrhalis* is also associated with lower levels of microbial biodiversity in the upper respiratory tract [35,39,49]. This suggests that a decrease in microbial diversity, together with the emergence of potential pathogens, might precede upper respiratory tract infections.

### 3.2 Lower respiratory tract infections

Pneumonia is one of the leading causes of mortality in children worldwide [55]. Although respiratory syncytial virus and influenza virus are described as the most common viral pathogens (29% and 17% of all episodes of pneumonia, respectively), most pneumonia-related deaths are caused by bacteria such as *S. pneumoniae* (33%) and *H. influenzae* type b (16%) [55].

There is a lack of knowledge regarding the role of respiratory microbiota in susceptibility to pneumonia. A recent study investigating the nasopharyngeal microbiota in children with pneumonia showed that viral pneumonia is associated with an increased abundance of *M. lacunata* in the upper respiratory tract, whilst non-viral (and therefore presumably bacterial) pneumonia is associated with a high abundance of *S. pneumoniae, H. influenzae*, and *M. catarrhalis* in comparison to healthy controls [56]. In line with previous studies investigating infectious diseases, pneumonia was associated with a lower microbial biodiversity [5,56]. Unfortunately, the authors could not identify health-associated bacteria, possibly as a result of a lack of statistical power.

### 3.3 Cystc flbrosis

CF is one of the most common lethal genetic diseases in Caucasians [57,58]. In CF, a mutation in the CF transmembrane conductance regulator (CFTR) gene results in altered ion transport affecting multiple body systems, including the respiratory tract. In the airways, CF causes hyperviscosity of secretions, impairing mucociliary clearance and promoting stasis and accumulation of mucus. This facilitates chronic respiratory infections that require frequent and chronic treatment with broad-spectrum antibiotics. Culture-dependent studies have identified a small number of bacteria as common CF pathogens in children, including *Pseudomonas aeruginosa, S. aureus, S. pneumoniae, H. influenzae*, and *Burkholderia cepacia* [57–59]. New culture-independent techniques have improved the knowledge on the complex microbial ecosystems in the airways of paediatric CF patients. A wide range of bacteria is now found to be associated with CF, although their role in the pathogenesis of respiratory diseases is still uncertain [60–62]. The microbial biodiversity of the CF lung has been shown to increase in the first few months of life, then peak during childhood with a predominance of *H. influenzae* and *S. aureus*, followed by a decrease in microbial diversity during the second decade of life when it is generally predominated by a few antibiotic-resistant pathogens including *P. aeruginosa* [58–60,63,64]. The decrease in microbial diversity and the presence of *P. aeruginosa* have been strongly associated with severity of disease [58,61,63–65]. Moreover, *P. aeruginosa* inversely correlates with lung function and microbial diversity [62–64,66,67]. It is believed that frequent antibiotic treatment plays an important role in the decrease in microbial biodiversity over time. Additionally, a correlation between diversity of the microbiota and CFTR genotype has been reported [63,64]. In summary, the CF lung microbiota is generally characterised by a decreased microbial biodiversity and an increased abundance of CF pathogens over time, which is likely to be influenced by multiple factors including repeated and chronic antibiotic treatment as well as chronic inflammation. The relationship between microbiota composition, inflammation and lung function deterioration deserves more in depth study—this information may help to predict the course of disease, and might help to treat acute and chronic infections with alternative methods.

### 3.4 Bronchopulmonary dysplasia

Bronchopulmonary dysplasia (BPD) is a lung disease characterised by inflammation and scarring that occurs in premature children. In infants born before 28 weeks of gestational age, 50–80% develop BPD; therefore, BPD accounts for the vast majority of cases of chronic lung disease in young infants [68]. The pathogenesis of BPD is complex and seems to be multifactorial. Several studies using new molecular techniques suggested that the (initial) composition of the microbial community may be associated with the development of BPD, for example, by affecting inflammation and/or chronic microbial infections [69–75]. A recent study in preterm infants (24–32 weeks of gestational age) showed that endotracheal aspirates of infants who developed BPD showed a less diverse respiratory microbiota in comparison to infants who did not develop BPD [76]. This study also showed that the microbiota composition of infants who would develop BPD was less stable over time and that it was characterised by an increased abundance of *Klebsiella* and *Staphylococcus* spp. Moreover bacterial presence in respiratory samples, regardless of the bacterial species, has been associated with BPD [69,70,73]. This may indicate a possible infectious aeiology.

Special interest has been paid to the presence of *Ureaplasma* spp. in the respiratory tract and the development of BPD/chronic lung disease [69,71,72,74]. The presence of *Ureaplasma* spp. appears to be associated with increased severity of the disease but also with other known risk factors for the development of BPD such as lower gestational age, lower birth weight and the ocurrence of chorioamnionitis, which makes it hard to unravel the direct cause and effect relationships [71,72,75,76].

### 3.5 Asthma

Asthma is a chronic inflammatory respiratory disorder mediated by a multitude of immune cell types and inflammatory mediators produced by mast cells, eosinophils, Th2 lymphocytes and interleukines [77]. Its exact aetiology and pathogenesis are still poorly understood. Recently, it has been postulated that respiratory bacteria might actually play a causative role in the pathogenesis of asthma itself, rendering it to be a chronic infectious disease rather than sterile inflammation [78,79].

A diverse microbial environment seems to enhance a more diverse human microbiota, thereby protecting against development of atopic diseases [80,81]. In line with this, several studies have demonstrated that early life antibiotic treatment (which causes a decrease in microbial biodiversity) is associated with asthma [82,83]. This is supported by research showing that asthmatic patients with higher microbial diversity may have a better lung function [84,85].

Similar to what is observed in adults [84–86], the respiratory microbiota of asthmatic or wheezing children is characterised by a high abundance of *Proteobacteria*, especially *H. influenzae* [86]. In other studies, members of *Staphylococcus, Enterobacteriaceae, Bacteroides*, and *Clostridium* spp. have also been associated with asthma [79,87]. Also, early life colonisation with *H. influenzae, M. catarrhalis*, and *S. pneumoniae* in healthy Danish children has been associated with persistent wheeze over time, which can even develop towards asthma after the first 5 years of life [88]. In line with this, a *Streptococcus-dominated* microbial profile appeared to be a strong asthma predictor, as recently shown in an Australian healthy birth cohort study [89]. Mode of birth delivery has been shown to affect the risk of developing asthma [90,91]; children born by Caesarean section were found to be more prone to develop asthma later in life than children born vaginally, which might be linked to early microbiota development that is significantly altered in infants born by caesarean section [20]. Also, antibiotic treatment with macrolides seems to be effective in the treatment of acute asthma exacerbations, especially steroid-resistant asthmatic children [88]. This might imply that bacterial colonisation is of key importance in the pathogenesis of asthma or that macrolide treatment renders an important immunomodulatory effect.

Of the large variety of risk factors that are associated with asthma, many are linked to the gastrointestinal microbiota [92]. An elaborate disquisition on how the gut microbiota may influence the respiratory tract microbiota goes beyond the scope of this review. However, a detailed summary of the literature available on this subject has been described by Bendiks etal. [93].

## 4. Discussion

This review provides a summary of the current literature on the relationship between the respiratory tract microbiota in children and respiratory diseases. The microbiota of the respiratory tract in children with (severe) respiratory diseases seems to differ from that of healthy children. During respiratory infections, colonisation with potential pathogens appears to be enhanced, suggesting a crucial step for infections to emerge. Moreover, loss of microbial biodiversity and a decrease in beneficial commensals are reported during respiratory infections. This might indicate that respiratory infectious diseases are related to microbial dysbiosis. However, due to a lack of longitudinal data, it remains unclear whether these changes in the microbiota are part of causative mechanisms, merely a consequence of respiratory diseases, or part of bystander phenomenon. Prospective cohort studies in combination with *in vitro* studies on mechanisms of effect are therefore of great importance in revealing the potential role of the microbiota in the pathogenesis of disease. Further well-designed longitudinal investigations are needed in order to gain more insights into the mechanisms behind microbiota variations and their effect on health. Currently, several initiatives for such studies have been put into effect by our group and others [94].

In addition, this review describes environmental factors that affect the composition and development of the respiratory microbiota. Mode of birth delivery, feeding type, use of antibiotics, and vaccinations might all together have an influence on respiratory microbiota development in early life. Longitudinal studies are required to gain more insights into these and other environmental factors that might influence the development of the respiratory microbiota and thereby susceptibility to respiratory diseases.

Microbiota studies are a relatively new field of research and therefore the number of studies regarding the respiratory microbiota in children and infectious respiratory diseases is still relatively small. Moreover, many studies use small participant groups, which makes it difficult to draw solid conclusions. One impediment in comparing data is the variation in age groups among different studies, since age itself has been shown to be an important determinant in the development of the microbiota. Also, geographical and seasonal influences might differ between studies. In addition, it can be difficult to compare studies because methodology (e.g. sample technique, study design, and tools used for analyses) and study populations vary between studies. Nevertheless, it is a very promising research field and new, larger (preferably longitudinal) studies are needed in order to clarify the exact role of the microbiota and other environmental determinants in paediatric health and disease. This is of major importance, as antibiotic resistance is on the rise and alternative treatment options are needed. Novel molecular research methods might enable us to design innovative ways of prevention and treatment of diseases, because by altering the microbiota of individuals it might be possible to intervene in pathways leading to disease. Illustrating this, there has been an increasing interest in the use of pre-and probiotics during the past decades. It is hypothesised that by promoting beneficial bacteria, diseases might be prevented or treated [95–97]. A meta-analysis of the effect of probiotics on treatment or prevention of respiratory infections showed a positive though modest effect [98]. The complexity and heterogeneity in microbial ecosystems between individuals means that a ‘one cure fits all’ strategy seems to be oversimplified.

## 5. Conclusion

In conclusion, this review shows that the respiratory microbiota of healthy children differs from that of children suffering from respiratory diseases. The latter group is mainly characterised by colonisation profiles containing high abundance of potential pathogens, a loss of microbial biodiversity, and a decrease of (beneficial) commensals. Prospective studies taking into account the temporal variability of the microbiota and comparing patients with healthy subjects, will provide a better understanding of the role of the microbiota in the dynamics of the pathogenesis of respiratory disease. This could provide the basis for new ways to prevent and treat respiratory diseases in children in the future.

## Search strategy and selection criteria

We identified references for this review by searches in different databases (PubMed, Embase, PiCarta and the Cochrane library), and references from relevant articles. The following MeSH headings were used: “Infant, Newborn”, “Infant”, “Child, Preschool”, “Child”, “Adolescent”, “Respiratory System”, “Respiratory Tract Diseases”, “Microbiome”, “Metagenome”, “Metagenomic”, “16s ribosomal rna”. In addition the following text words were used: “newborn*”, “infan*”, “child*”, “adolescen*”, “pediatric*”, “paediatric*”, “neonat*”, “airway*”, “lung*”, “nasopharyn*”, “respiratory”, “pulmonary”, “sinus*”, “otiti*”, “pharyngitis”, “bronchitis”, “bronchiolitis”, “pneumonia”, “bronchopulmonary dysplasia”, “chronic lung disease”, “asthma*”, “astma*”, “cystic fibrosis”, “microbiome*”, “metagenome*”, “microbiota*”, “pyrosequencing”, “culture independent”, “culture-independent”, “16 s”, “16s”, “microbial communit*”, “bacterial communit*”, “bacterial carriage”, “viral carriage”, “viral communities”, “microflora”.

This search resulted in 896 references, which were all further assessed by reading the title and abstract. Only articles published in English were included. We further assessed the references of all selected publications. The final reference list was generated on the basis of relevance to the topics covered in this review.
